# Supplementary material for: Cutting-Edge Sensor Design: MIP Nanoparticle-Functionalized Nanofibers for Gas-Phase Detection of Limonene in Predictive Agriculture
Source: Polymers (Basel). 2025 Jan 25;17(3):326. doi: 10.3390/polym17030326 (PMC11820196; doi:10.3390/polym17030326)
Supplement: Supplementary file 1 [file polymers-17-00326-s001.zip › polymers-3426183-supplementary.pdf]

POLYMERS SUPPLEMENTARY MATERIALS

"Cutting-Edge Sensor Design: MIP Nanoparticle-Functionalized Nanofibers for Gas-Phase Detection of Limonene in Predictive Agriculture"

Fabricio Nicolas Molinari, Marcello Marelli, Enrico Berretti, Simone Serrecchia, Roxana Coppola, Fabrizio De Cesare and Antonella Macagnano

VOC sensors provide a non-invasive real-time plant health monitoring method by detecting biochemical signals associated with plant biotic and abiotic stress and phenological states. These sensors can optimise agricultural inputs like water and agrochemicals, thereby reducing environmental pollution and natural resource depletion. However, the practical application of these sensors in agriculture is still under development, with challenges related to sensitivity, selectivity, and integration into existing agricultural systems.

Table S1. Comparison of the sensing features and the pros and cons of recently developed sensors for VOC from plants.

Classification	Sensing category	Sensing mechanisms	Benefits	Shortcomings	Applications
EN (CC); SS (LS)	EC	EV	High selectivity [1]; fast response time [1]; room temperature operation [1]]; low power consumption [2]; low cost [1,2]; low limit of detection [1]; tolerant to environmental factors [1]	Limited operational lifetime [2]; poor baseline stability [2]	Phytopathogen detection [3]
	Gravimetric (QCM)	MV+EV	Work at room temperature with high sensitivity [4]; Long lifetime [5]	Poor reproducibility [4]; low sensitivity [5]; sensitive to environmental factors [5]; limitations in accuracy at low concentrations [6]	Fruit ripeness and freshness monitoring [7] virus detection [8]

Classification	Sensing category	Sensing mechanisms	Benefits	Shortcomings	Applications
	Optical	OV	Disposable [4]; portable [4] [9]; fast response [4]; robust [4]	Sensitive to humidity [4] [9]; Complexity in miniaturization [9]; requires precise calibration [9]	Phytopathogen detection [3]; wood drying [10]
	CP	EV	High sensitivity [3]; low cost [3]; short response time [3,4]; low energy consumption [3]; portable [3]	Unstable [3]; poor selectivity [3]; sensitive to environmental factors [3]	Pests and phytopathogen detection [11]
	MOS	EV	High sensitivity [4]; cross-sensitivity [12]; fast [4,13]; reliable [13]; low cost [13]; non-destructive [13]	High temperature [4]; low selectivity [14]; sensitive to environmental factors [14,15]	Pests and phytopathogen detection [11,16]
SS (LS)	FAIMS	IC	Operates at atmospheric pressure [17]; inexpensive [17]; high sensitivity[18]; tolerance to environmental conditions [18]; portable [18] [19,20]	Lower performance [17]; lower accuracy [18]	Phytopathogen detection [11]
SS (LS)	Biosensing	BV	Real-time [4]; high specificity [4]	Unstable [4]; sensitive to pH [4]; sensitive to environmental factors [4];	Phytopathogen detection [21] [22]; plant stress [23]

Classification	Sensing category	Sensing mechanisms	Benefits	Shortcomings	Applications
SS (LS)	Wearable	EV	Remarkable sensitivity and selectivity in detecting target VOC [24]; possible multi-target sensing [24] [25], high resolution data with minimal interferences to the plant[25]	Stability of detection may diminish as the complexity of compounds to be identified or classified increases [24]; require direct attachment to the plant [26] ; still at an early stage development	Pests and diseases detection [24] [25], abiotic stress [24,25], phytometric analysis [25]

Acronyms:
LS = Laboratory scale
CS = Commercial scale
EN = Electronic noses (E-noses)
SS = Specific sensors
EC = Electrochemical
CP = Conductive polymers
MOS = Metal oxide semiconductors
FAIMS = Field asymmetric ion mobility spectrometry
EV = Electric variation
MV = Mass variation
OV = Optical (colourimetric) variation
BV = Biological variation (cells, enzymes, antibodies, ..)
IC = Ion current

Rutolo, M.F.; Clarkson, J.P.; Covington, J.A. The use of an electronic nose to detect early signs of soft-rot infection in potatoes. *Biosyst. Eng.* **2018**, *167*, 137–143

References

1. Venkatasetty, H.V. Electrochemical Amperometric Gas Sensors for Environmental Monitoring and Control. *SAE Technical Paper*, **1990**, 901296, https://doi.org/10.4271/901296 July 1 1990.
2. Rutolo, M.F.; Clarkson, J.P.; Covington, J.A. *Biosyst. Eng.* **2018**, *167*, 137–14, doi: 10.1016/j.biosystemseng.2018.01.001.
3. MacDougall, S.; Bayansal, F.; Ahmadi, A. Emerging Methods of Monitoring Volatile Organic Compounds for Detection of Plant Pests and Disease. *Biosensors (Basel)* **2022**, *12*, 239, doi:10.3390/bios12040239.
4. Cui, S.; Ling, P.; Zhu, H.; Keener, H. Plant Pest Detection Using an Artificial Nose System: A Review. *Sensors* **2018**, *18*, 378, doi:10.3390/s18020378.
5. Liu, X.; Cheng, S.; Liu, H.; Hu, S.; Zhang, D.; Ning, H. A Survey on Gas Sensing Technology. *Sensors* **2012**, *12*, 9635–9665, doi:10.3390/s120709635.
6. Ali, S.B.; Ghatak, B.; Debabhuti, N.; Pal, S.; sharma, prolay; Tudu, B.; Bhattacharyya, N.; Bandyopadhyay, R. Determination of Alpha-Myrcene Volatile in Mango by Quartz Crystal Microbalance Sensor. *IEEE Sens J* **2019**, *19*, 893–900, doi:10.1109/JSEN.2018.2879539.
7. Zhou, Q.; Zheng, C.; Zhu, L.; Wang, J. A Review on Rapid Detection of Modified Quartz Crystal Microbalance Sensors for Food: Contamination, Flavour and Adulteration. *TrAC Trends in Analytical Chemistry* **2022**, *157*, 116805, doi:10.1016/j.trac.2022.116805.

8. Dickert, F.L.; Hayden, O.; Bindeus, R.; Mann, K.-J.; Blaas, D.; Waigmann, E. Bioimprinted QCM Sensors for Virus Detection? Screening of Plant Sap. *Anal Bioanal Chem* **2004**, *378*, 1929–1934, doi:10.1007/s00216-004-2521-5.
9. Choi, Y.S.; Son, W.K.; Kwak, H.; Park, J.; Choi, S.; Sim, D.; Kim, M.G.; Kimm, H.; Son, H.; Jeong, D.H.; et al. Real-Time Monitoring of Volatile Organic Compound-Mediated Plant Intercommunication Using Surface-Enhanced Raman Scattering Nanosensor. *Advanced Science* **2024**, doi:10.1002/advs.202412732.
10. Nikoutadbir, A.; Tarmian, A.; Mohtasebi, S.S.; Abdulkhani, A. Emission of Volatile Organic Compounds from Heat-Treated Scots Pine Wood as Affected by Wood Drying Method: Results Obtained with Olfactory Machine and Headspace Gas Chromatography-Mass Spectrometry. *Drying Technology* **2023**, *41*, 577–589, doi:10.1080/07373937.2022.2109160.
11. MacDougall, S.; Bayansal, F.; Ahmadi, A. Emerging Methods of Monitoring Volatile Organic Compounds for Detection of Plant Pests and Disease. *Biosensors (Basel)* **2022**, *12*, 239, doi:10.3390/bios12040239.
12. Sun, Y.; Wang, J.; Cheng, S.; Wang, Y. Detection of Pest Species with Different Ratios in Tea Plant Based on Electronic Nose. *Annals of Applied Biology* **2019**, *174*, 209–218, doi:10.1111/aab.12485.
13. Kresnawaty, I.; Mulyatni, A.S.; Eris, D.D.; Prakoso, H.T.; Tri-Panji; Triyana, K.; Widiastuti, H. Electronic Nose for Early Detection of Basal Stem Rot Caused by Ganoderma in Oil Palm. *IOP Conf Ser Earth Environ Sci* **2020**, *468*, 012029, doi:10.1088/1755-1315/468/1/012029.
14. Dey, A. Semiconductor Metal Oxide Gas Sensors: A Review. *Materials Science and Engineering: B* **2018**, *229*, 206–217, doi:10.1016/j.mseb.2017.12.036.
15. Kataria, S.; Chandel, M.; Kumar, P.; Palanisami, M.; Moun, N.; Kanagarajan, S.; Shanmugam, V. Irrigation-Friendly Sensor to Manage Drought in Crops through Carbon-Based Signature Volatile Sensing. *Sens Actuators B Chem* **2024**, *403*, 134975, doi:10.1016/j.snb.2023.134975.
16. Gan, Z.; Zhou, Q.; Zheng, C.; Wang, J. Challenges and Applications of Volatile Organic Compounds Monitoring Technology in Plant Disease Diagnosis. *Biosens Bioelectron* **2023**, *237*, 115540, doi:10.1016/j.bios.2023.115540.
17. Rutolo, M.; Covington, J.; Clarkson, J.; Iliescu, D. Detection of Potato Storage Disease via Gas Analysis: A Pilot Study Using Field Asymmetric Ion Mobility Spectrometry. *Sensors* **2014**, *14*, 15939–15952, doi:10.3390/s140915939.
18. Costanzo, M.T.; Boock, J.J.; Kemperman, R.H.J.; Wei, M.S.; Beekman, C.R.; Yost, R.A. Portable FAIMS: Applications and Future Perspectives. *Int J Mass Spectrom* **2017**, *422*, 188–196, doi:10.1016/j.ijms.2016.12.007.
19. Sinha, R.; Khot, L.R.; Schroeder, B.K. FAIMS Based Sensing of Burkholderia Cepacia Caused Sour Skin in Onions under Bulk Storage Condition. *Journal of Food Measurement and Characterization* **2017**, *11*, 1578–1585, doi:10.1007/s11694-017-9537-y.
20. Sinha, R.; Khot, L.R.; Schroeder, B.K.; Si, Y. Rapid and Non–Destructive Detection of Pectobacterium Carotovorum Causing Soft Rot in Stored Potatoes through Volatile Biomarkers Sensing. *Crop Protection* **2017**, *93*, 122–131, doi:10.1016/j.cropro.2016.11.028.
21. Li, Z.; Paul, R.; Ba Tis, T.; Saville, A.C.; Hansel, J.C.; Yu, T.; Ristaino, J.B.; Wei, Q. Non-Invasive Plant Disease Diagnostics Enabled by Smartphone-Based Fingerprinting of Leaf Volatiles. *Nat Plants* **2019**, *5*, 856–866, doi:10.1038/s41477-019-0476-y.
22. Hassan, M.H.; Omar, A.M.; Daskalakis, E.; Mohamed, A.A.; Boyd, L.A.; Blanford, C.; Grieve, B.; Bartolo, P.JDS. Multi-Layer Biosensor for Pre-Symptomatic Detection of Puccinia Strifformis, the Causal Agent of Yellow Rust. *Biosensors (Basel)* **2022**, *12*, 829, doi:10.3390/bios12100829.
23. Fang, Y.; Ramasamy, R.P. A Portable Electrochemical System for Plant Volatile Detection. *ECS Trans* **2018**, *85*, 1359–1367, doi:10.1149/08513.1359ecst.
24. Lee, S.; Kim, J.; Kim, D.; Park, H.; Myoung, S.; Han, J.; Park, C.; Kim, Y.; Choi, C.; Lee, G. Wearable Volatile Organic Compound Sensors for Plant Health Monitoring. *Adv Sustain Syst* **2024**, *8*, doi:10.1002/adsu.202300634.

25. Kuruppuarachchi, C.; Kulsoom, F.; Ibrahim, H.; Khan, H.; Zahid, A.; Sher, M. Advancements in Plant Wearable Sensors. *Comput Electron Agric* **2025**, *229*, 109778, doi:10.1016/j.compag.2024.109778.
26. Lee, S.; Kim, J.; Kim, D.; Park, H.; Myoung, S.; Han, J.; Park, C.; Kim, Y.; Choi, C.; Lee, G. Wearable Volatile Organic Compound Sensors for Plant Health Monitoring. *Adv Sustain Syst* **2024**, *8*, doi:10.1002/adsu.202300634.
